# Effect of intra-articular injection of a hyaluronic acid-alendronate conjugate on post-traumatic osteoarthritis induced by destabilization of the medial meniscus in rats

**DOI:** 10.1038/s41598-023-46965-5

**Published:** 2023-11-24

**Authors:** Anna Scanu, Roberto Luisetto, Mauro Pavan, Cristian Guarise, Riccardo Beninatto, Chiara Giraudo, Francesca Galuppini, Vanni Lazzarin, Vincenza Guzzardo, Gianmaria Pennelli, Devis Galesso, Stefano Masiero

**Affiliations:** 1 Rehabilitation Unit, Department of Neuroscience-DNS, University of Padova, 35128 Padua, Italy; 2 Department of Surgery, Oncology and Gastroenterology—DISCOG, University of Padova, 35128 Padua, Italy; 3 R&D–Discovery, Fidia Farmaceutici SpA, Via Ponte della Fabbrica, 3/a, 35031 Abano Terme, Italy; 4 Nuclear Medicine Unit, Department of Medicine—DIMED, Padova University Hospital, 35128 Padua, Italy; 5 Surgical Pathology Unit, Department of Medicine—DIMED, University of Padova, 35128 Padua, Italy

**Keywords:** Osteoarthritis, Pharmaceutics

## Abstract

Osteoarthritis (OA) is a chronic degenerative joint disease characterized by pain and cartilage damage. Intra-articular (i.a) viscosupplementation with hyaluronic acid (HA) is frequently used for the management of OA. Preclinical studies have reported that bisphosphonates (BPs) may have a therapeutic potential to slow down or reverse the progression of OA. Among these, alendronate (ALN) has demonstrated chondroprotective effects in both in vitro and vivo experiments. This study evaluated the effects of a novel alendronate-hyaluronic acid (ALN-HA) conjugate on an OA in vivo model induced by medial meniscus destabilization (DMM). DMM surgery was performed on the knees of Sprague Dawley rats that received, after four weeks, one intra-articular (i.a.) injection of: (1) ALN-HA; (2) HA; (3) sodium chloride (NaCl). Sham-operated rats were used as control. Allodynia was assessed by Von Frey test. Joint degeneration was evaluated eight weeks after treatment by micro-computed tomography (micro-CT), histology, and immunohistochemistry. Collagen cross-linked C-telopeptides (CTX-I and CTX-II) serum levels were determined by ELISA. Paw withdrawal threshold increased in ALN-HA group when compared to rats treated with NaCl or HA. Micro-CT did not show differences between ALN-HA, HA and NaCl groups. ALN-HA injection produced significant improvements in articular cartilage degeneration showing an OARSI score lower than those of HA and NaCl, and reduced matrix metalloproteinase (MMP)-13, MMP-3, interleukin-6, vascular endothelial growth factor and Caspase-3 expression. CTX-I was reduced after ALN-HA treatment when compared to NaCl. Our results indicate that i.a. use of ALN after conjugation with HA limits OA development and progression in the rat DMM model, and may lead to the development of novel therapeutic strategies in OA management.

## Introduction

Osteoarthritis (OA) is the most common chronic degenerative joint diseases, with high incidence and prevalence which are expected to continue to increase worldwide^1–3^. OA is characterized by joint swelling, degradation and loss of joint cartilage, changes in bone architecture and mineralization within the subchondral bone, inflammation and pain^4,5^. Poorly managed OA can lead to limited joint function, reduce quality of life and induce significant disability^6^.

Currently, treatments for OA aim to reduce symptoms by a combination of non-pharmacological and pharmacological approaches. Conventional pharmacological treatments (e.g. non-steroidal anti-inflammatory drugs and opioids) are principally used to reduce pain, but these are not sufficient to effectively treat OA and are often associated with side effects following long-term use. In addition, approved drugs that exert both analgesic and chondroprotective effect are lacking^7–9^. Direct intra-articular (i.a.) injections of medications are commonly used to increase the availability of a drug in the joint. In this context, viscosupplementation with hyaluronic acid (HA) has demonstrated to be safe and effective in restoring the synovial fluid (SF) homeostasis and protecting articular cartilage from mechanical damage^10–12^. Furthermore, different reports have indicated that HA can exert also analgesic and anti-inflammatory effects^13,14^. However, controversies exist regarding its efficacy and safety, as evidenced by discordancies in the guidelines and recommendations related to the use in clinical practice^15,16^. On the other hand, no distinction is made among different HA sources, MWs and chemical modifications^17,18^.

In recent years, bisphosphonates (BPs), a class of drugs commonly used to treat osteoporosis, have been proposed as a potential disease-modifying treatment for OA, due to their ability to inhibit osteoclast-mediated bone loss that may have benefits on subchondral bone and cartilage. In this context, preclinical studies have shown that BPs may have a therapeutic potential to delay or reverse the progression of OA by a positive subchondral bone conservation, fewer biomarker alterations and an anti-inflammatory effect^19^. However, randomized controlled trials in OA patients report mixed results in pain improvement^20–22^. Among BPs, alendronate (ALN) has shown interesting results, reducing osteophyte formation and preventing early trabecular bone loss and cartilage degeneration in rat and mouse models of post-traumatic OA^23–25^. In addition, ALN exhibited a chondroprotective effect and suppressed the expression of metalloproteinases and cytokines, including matrix metalloproteinase (MMP)-13, MMP-9, interleukin (IL)-1β, collagen-10 (COLX), transforming growth factor beta (TGFβ), vascular endothelial growth factor (VEGF), in both in vitro and vivo experiments^26–31^. Interestingly, similar results and absence of cytotoxicity have been demonstrated in in vitro experiments for a novel hyaluronan conjugate with a covalent and hydrolysable linker between HA and ALN (ALN-HA)^32^. Additionally, the macromolecular ALN-HA conjugate presented a slow-release rate of the bisphosphonate, suggesting an extended joint retention of ALN, which otherwise would be quickly removed in vivo due to the 160 kDa cut-off of a healthy synovial membrane^33,34^.

The aim of this study was to explore the effect of a single administration of the chemical ALN-HA conjugate, compared to vehicle or HA, on the improvement of joint structures and pain in rats with knee OA induced by destabilization of the medial meniscus (DMM) surgery.

## Methods

### Preparation of the alendronate conjugate-hyaluronic acid (ALN-HA)

Synthesis and characterization of the chemical conjugate between ALN and 700 kDa HA were previously described^32^. Briefly, ALN-HA conjugate was prepared by mixing a solution of alendronate tetrabutylammonium salt (ALN-TBA) and HA tetrabutylammonium salt (HA-TBA) in presence of 2-chloroethyl 1H-imidazole-1-carboxylate, in anhydrous dimethylsulfoxide (DMSO). To prepare ALN-HA, ALN-TBA (18.6 g; 0.04 mol) was treated with a solution of 2-chloroethyl 1H-imidazole-1-carboxylate (27.5 g; 0.14 mol) in anhydrous DMSO (0.2 L) at 40 °C for 24 h under vigorous stirring. Then, the reaction mixture was added to a solution of HA-TBA (Fidia Farmaceutici S.p.A.—MW 700 kDa, 6.80 g; 0.01 mol) in anhydrous DMSO (0.5 L) and stirred at 40 °C for 48 h. The product was precipitated by adding slowly a saturated solution of sodium bromide (67 mL) and ethanol (1 L). Finally, the product was recovered by filtration, solvated in UPW (500 mL), then purified by an ultrafiltration cassette (Spectra/Por®, MWCO 20 kDa) against UPW at pH = 6 for 6 h and freeze-dried, affording the product as a white fluffy sponge (6.67 g, yield 97%). The chemical characterization of ALN-HA, conducted as previously described revealed a functionalization degree of 6.2% mol/mol (ALN vs HA repeat unit)^32^.

### Expertimental design

**Figure 1:**
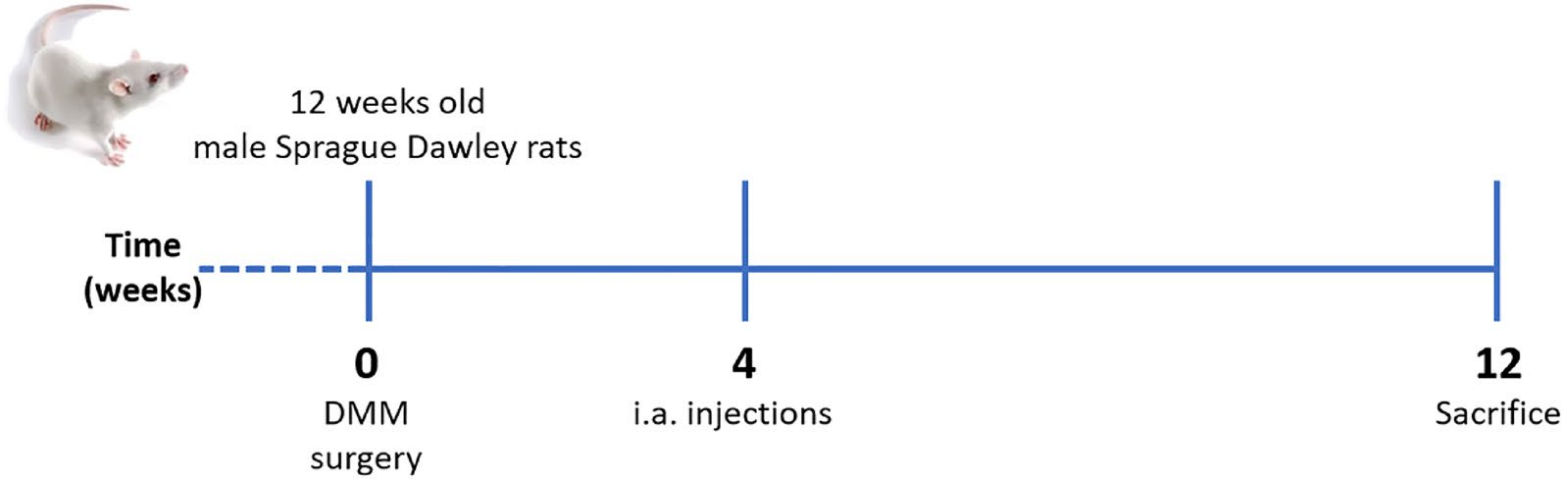
Experimental scheme. Osteoarthritis (OA) was induced by surgical destabilization of the medial meniscus (DMM) on the right knee of Sprague Dawley. A group of animals underwent surgery but without DMM (Sham). For weeks after DMM surgery rats received one i.a. injections of: (i) NaCl, (ii) alendronate conjugate-hyaluronic acid, and (iii) hyaluronic acid. Rats were euthanized 8 weeks after i.a. injection (12 weeks after surgery).

The experiments were conducted on male wild-type Sprague Dawley rats at 12 weeks of age that weighed 280–320 g. All animals were housed individually in polycarbonate cages, maintained on a standard laboratory diet and had free access to water. The study was performed in accordance with relevant guidelines and regulations and adhere to the ARRIVE guidelines (https://arriveguidelines.org/). All experimental procedures were approved by the Padova University Animal Ethic Committee and Italian Ministry of Health (Rome, Italy) registered under #337/2020-PR. An a priori sample size calculation (one-way ANOVA: effect size = 0.5; 1−β = 0.80; α = 0.05) was performed in order to determine the number of animals needed for the study, and found that 11 rats per group (total number = 44) was adequate. DMM was performed according to the method of Glasson et al. on the right knee of anesthetized rats, while a group of animals underwent surgery but without DMM (Sham)^35^. Briefly, rats were anesthetized, shaved and a longitudinal incision was made over the distal patella to the proximal tibial plateau in the right knee joint. Following a medial capsular incision and displacement of the knee extensor muscles, the medial meniscotibial ligament (MMTL) was identified and transected. After replacement of the extensor muscles, the medial capsular incision and the skin were sutured. A sham operation was performed using the same procedure without MMTL transection. Four weeks after DMM surgery, the animals were randomized into 3 groups receiving one i.a. injections of: (1) 50 µL of 0.9% NaCl sterile solution, (2) 50 µL of ALN-HA conjugate, resuspended at 10 mg/mL in phosphate buffered saline (PBS) pH 7.2, reaching a final ALN concentration of 1.5 mM, and (3) 50 µL of 700 kDa HA sodium salt (Fidia Farmaceutici SpA), previously formulated at 10 mg/mL in PBS pH7.2 (Fig. 1). Before the i.a. injection, the rats were anesthetized, and the right knee and surrounding area were shaved and cleaned with antiseptic solution (povidone iodine 10%). Injections were carried out using 27 gauge needle through the anterolateral portal with the knee flexed to 90°.

Rats were maintained in individual cages during the experiment to avoid confounders.

### Clinical evaluations

The rats were monitored daily for general health. Body weight was recorded before surgery, daily for the first week and once a week until sacrifice. Excessive body weight loss or signs of excessive suffering were considered criteria for excluding animals from the experimental design. If present, synovial fluid (SF) was aspirated from the knee and analyzed to determine the volume and degree of inflammation by a total leukocyte count^36^.

### Mechanical allodynia

Mechanical allodynia was quantified weekly by measuring the hind paw withdrawal response by an electronic Von Frey anesthesiometer (ugo basile s.r.l., Varese, Italy). Briefly, the animals were placed in plexiglass boxes equipped with a metallic meshy floor and were allowed to habituate themselves to their environment for 15 min before the test. The von Frey rigid tip monofilament was applied to the plantar surface of the right hind paws and the withdrawal threshold was automatically recorded by the device. The withdrawal threshold was evaluated by applying forces ranging from 0 to 200 g with a probe of 0.2 mm diameter. The measure was repeated 5 times with an interval of 180 s and then expressed as a mean. All recordings were conducted by a blinded investigator.

### Collection and processing of samples

Eight weeks after i.a. injection (1 weeks after surgery) rats were sacrificed by excess anesthesia with sevoflurane and paw samples were collected and fixed in 4% (w/v) paraformaldehyde. In addition, blood was harvested by cardiac puncture and centrifuged at 1500 rpm for 10 min at 20 °C (ALC PK 130 centrifuge, rotor No. T535) to obtain serum.

### Micro-computed tomography analysis

Each sample was placed in a cylindrical polyethylene container and analyzed by an ex-vivo Micro-CT Skyscan 1275 scanner (Bruker, Kontich, Belgium). The following Micro-CT parameters were applied: 65 kV, 80 μA, 1 mm aluminum filter, and 21 μm voxel size. The acquired raw data were reconstructed and analyzed with the N-Recon and CT-An software, respectively (Bruker microCT, Kontich, Belgium). To compute the bone mineral density (mg/mm^3^) one sample of saline solution and two samples of CaHA of 0.25 and 0.75 g/cm^3^ were acquired and reconstructed applying the same parameters. For each sample, a region of interest (ROI) of the trabecular and cortical bone were performed in the distal femoral and proximal tibial epiphyses. Then trabecular bone volume (BV, %), trabecular thickness (μm), trabecular separation (μm), and bone mineral density (mg/mm^3^) of the bone marrow were computed. For the cortical bone, cortical thickness (μm) and tissue mineral density (mg/mm^3^) were extracted. For each parameter the mean value was computed. All assessments were carried out by a blinded investigator.

### Histological analysis

Following fixation, right hind paw samples were decalcified in a solution of formic acid, nitric acid, and distilled water for 24 h, and embedded in paraffin. Then, 5 μm frontal sections were taken through the entire joint and stained with Hematoxylin–Eosin or Safranin O/Fast Green for histological analysis. Slides were examined by two blinded independent investigators using a Leica DM4000B microscope equipped with a Leica DFC420 camera. Degradation of femoral condyle and tibial plateau cartilage was quantified with the OARSI score^37^, which is the primary outcome of the study. In addition, a five-tier system (0–4) was used to evaluate the proteoglycan content (0 = no loss of proteoglycan staining relative to a normal control, 1 = minimal loss, 2 = mild loss, 3 = moderate loss and 4 = total loss of pro-teoglycan staining)^38^. Three slides per joint were used to generate each data point.

### Immunohistochemistry

Immunohistochemical (IHC) staining were performed automatically on a Leica BondTM-MAX system using the Bond Polymer Refine Detection kit (Leica Biosystems, Newcastle upon Tyne, UK). Formalin-fixed paraffin-embedded 4 μm-thick sections were pretreated using heat mediated antigen retrieval with sodium citrate buffer (pH6, epitope retrieval solution 1, Leica) for 30 min a 99 °C. The following primary antibodies were used: anti-MMP-3 (clone 1B4, Santa Cruz Biotechnology, Inc., Dallas, TX, USA; 1:50 dilution), anti-VEGF (clone C-1, Santa Cruz Biotechnology; 1:250 dilution), anti-caspase (Casp)-3 (clone E-8, Santa Cruz Biotechnology; 1:100 dilution), anti-MMP-13 (rabbit polyclonal, Proteintech Group, Inc., Rosemont, USA; 1:200 dilution), anti-A disintegrin and metalloproteinase with thrombospondin motifs (ADAMTS)-5 (rabbit polyclonal, Biorbyt Ltd, Cambridge,UK; 1:50 dilution), anti-IL-6 (rabbit poly-clonal, Biorbyt; 1:200 dilution), and anti-COL10A1 (rabbit polyclonal, Biorbyt; 1:100 dilution). Staining was visualized with 3,3′-diaminobenzidine (DAB) and the slides were lightly counterstained with hematoxylin. Appropriate positive and negative controls were used. The mean percentage of positive staining cells was determined by counting cells at 10 different fields observed under 400X magnification using a light microscope by two blinded independent investigators. For each region of interest a score of 0–4 was determined, where 0 = negative; 1 =  < 25% of cells with positive staining; 2 =  ≥ 25% and < 50% of cells with positive staining; 3 =  ≥ 50% and < 75% of cells with positive staining; 4 = more than ≥ 75% of cells with positive staining were assigned for MMP-3, MMP-13, IL-6, VEGF, ADAMTS-5 and Casp-3; and where 0 = negative; 1 =  < 10% of cells with positive staining; 2 =  ≥ 10% and < 25% of cells with positive staining; 3 =  ≥ 25% and < 50% of cells with positive staining; 4 = more than ≥ 50% of cells with positive staining were assigned for COL10A1.

### Serum analysis

Type I and II collagen cross-linked C-telopeptides (CTX-I and CTX-II) serum levels were measured by commercially available enzyme immunoassay ((Wuhan Fine Bio-tech Co., Wuhan, China).

### Statistical analysis

Data are expressed as the mean ± SD. Statistical differences between experimental groups were assessed by nonparametric one-way analysis of variance (ANOVA), as the variables were not normally distributed (Shapiro–Wilk normality test). Multiple comparisons were performed by Dunnett’s test. GraphPad Prism software 5.0 (Version 5.01, GraphPad Software, Inc., San Diego, CA, USA) was used for analysis, and a p-value < 0.05 was considered significant.

### Ethics approval and consent to participate

The animal study protocol was approved by the Padova University Animal Ethic Committee and Italian Ministry of Health (protocol code 337/2020-PR, date of approval 22 April 2020) for studies involving animals.

## Results

### Clinical assessment

All the animals reached the end of the study and no one showed clinical signs related to the onset of other pathologies. One week after DMM surgery, 5 rats displayed swollen knee joint, from which SF was collected (50 μl in three cases and 100 μL in the other two cases). All synovial fluids were non-inflammatory (total leukocyte count: < 1000 cells/mm^3^). No change in body weight was observed between groups (data not shown).

### Effect of ALN-HA derivative in mechanical allodynia

**Figure 2:**
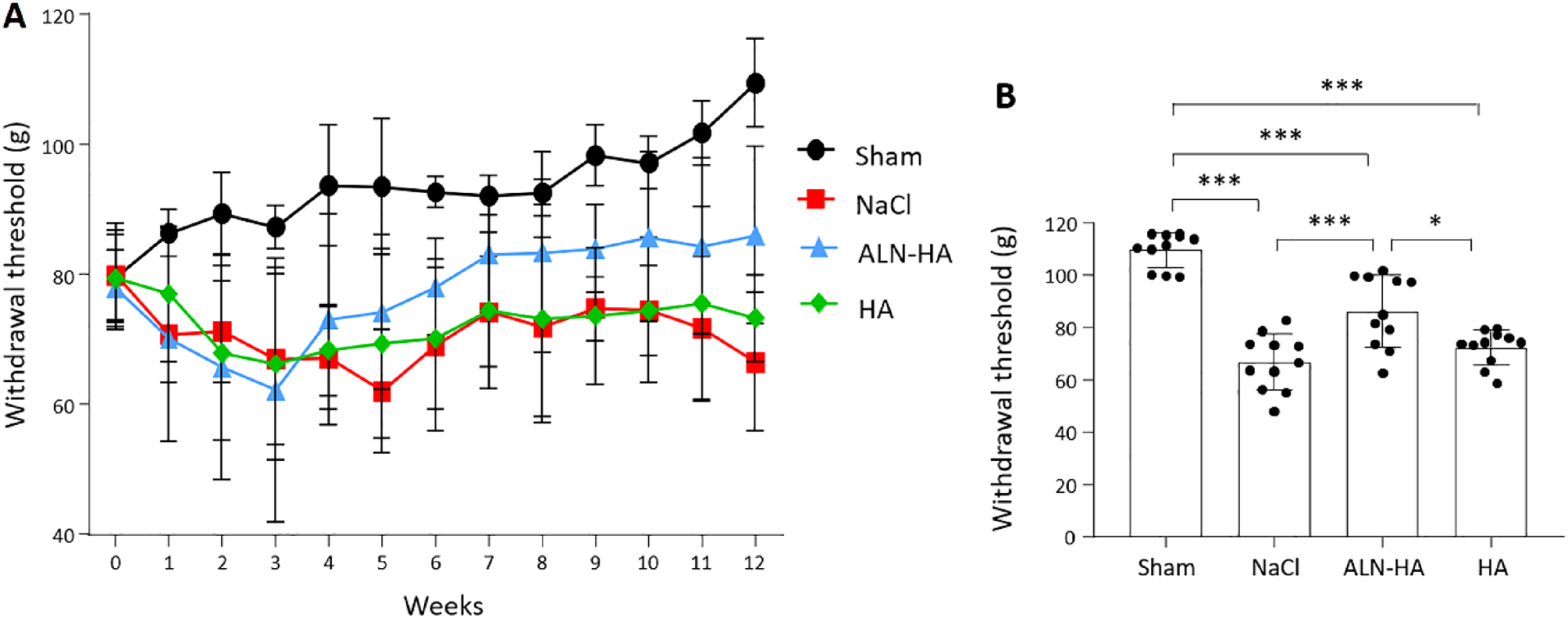
Mechanical allodynia assessed by von Frey test. Paw withdrawal threshold in response to von Frey filament stimulation (**A**) at several time points and (**B**) at the end of the experiments after DMM surgery in NaCl, alendronate conjugate-hyaluronic acid (ALN-HA), and HA treated-groups, and in sham-operated rats. Results are presented as mean ± SD of 11 rats per group. *p < 0.05, ***p < 0.001.

Mechanical allodynia assessment demonstrated that withdrawal thresholds for the sham group were the highest throughout the whole time course of the experiment (Fig. 2A). All animals subjected to surgery developed allodynia after 1 week and the recorded values decreased during the first 3 weeks. From the fourth week, the withdrawal threshold remained low, did not change significantly over time and was similar in rats treated with NaCl or HA, while it increased for the ALN-HA group. At the end of the experiment, the mean right hind paw withdrawal thresholds in the ALN-HA-treated animals were significantly higher than those recorded in the NaCl- or HA-injected groups (29.29% and 17.43%, respectively) (Fig. 2B).

### Micro-CT

The results obtained by micro-CT analysis are reported in Table 1. Micro-CT analysis found significant changes between Sham and NaCl in trabecular BV (decrease, p < 0.05), trabecular space (increase of, p < 0.05), cortical bone thickness (decrease, p < 0.05) and cortical bone BMD (decrease, p < 0.01). Only a slight increase in trabecular BV (11.58%), trabecular thickness (6.11%) and cortical bone thickness (6.81%) was observed in the samples from rats treated with ALN-HA when compared with the NaCl group. However, no significant changes were observed between NaCl and ALN-HA or HA, and between ALN-HA and HA for any of the parameters considered.

**Table 1:** Results of trabecular and cortical micro-CT parameters measured in sham and treated knee joints with NaCl, ALN-HA, or HA at 12 weeks after DMM surgery.

		SHAM	NaCl	ALN-HA	HA
Trabecular	BV (%)	55.30 ± 6.12	45.64 ± 9.08*	50.92 ± 8.07	48.74 ± 6.26
	Thickness (μm)	156.01 ± 10.22	145.86 ± 9.15	154.75 ± 8.92	149.12 ± 8.50
	Separation (μm)	209.04 ± 31.51	251.78 ± 35.43*	230.57 ± 42.94	226.52 ± 28.64
	BMD (mg/mm^3^)	0.69 ± 0.07	0.64 ± 0.06	0.64 ± 0.07	0.66 ± 0.08
Cortical	Thickness (μm)	281.47 ± 16.53	257.23 ± 25.20*	274.80 ± 24.29	267.83 ± 18.85
	BMD (mg/mm^3^)	0.97 ± 0.01	0.94 ± 0.01***	0.95 ± 0.02*	0.94 ± 0.01**

*p < 0.05, **p < 0.01, ***p < 0.001 vs sham group.

### Effect of ALN-HA in degenerative changes of joint tissues

**Figure 3:**
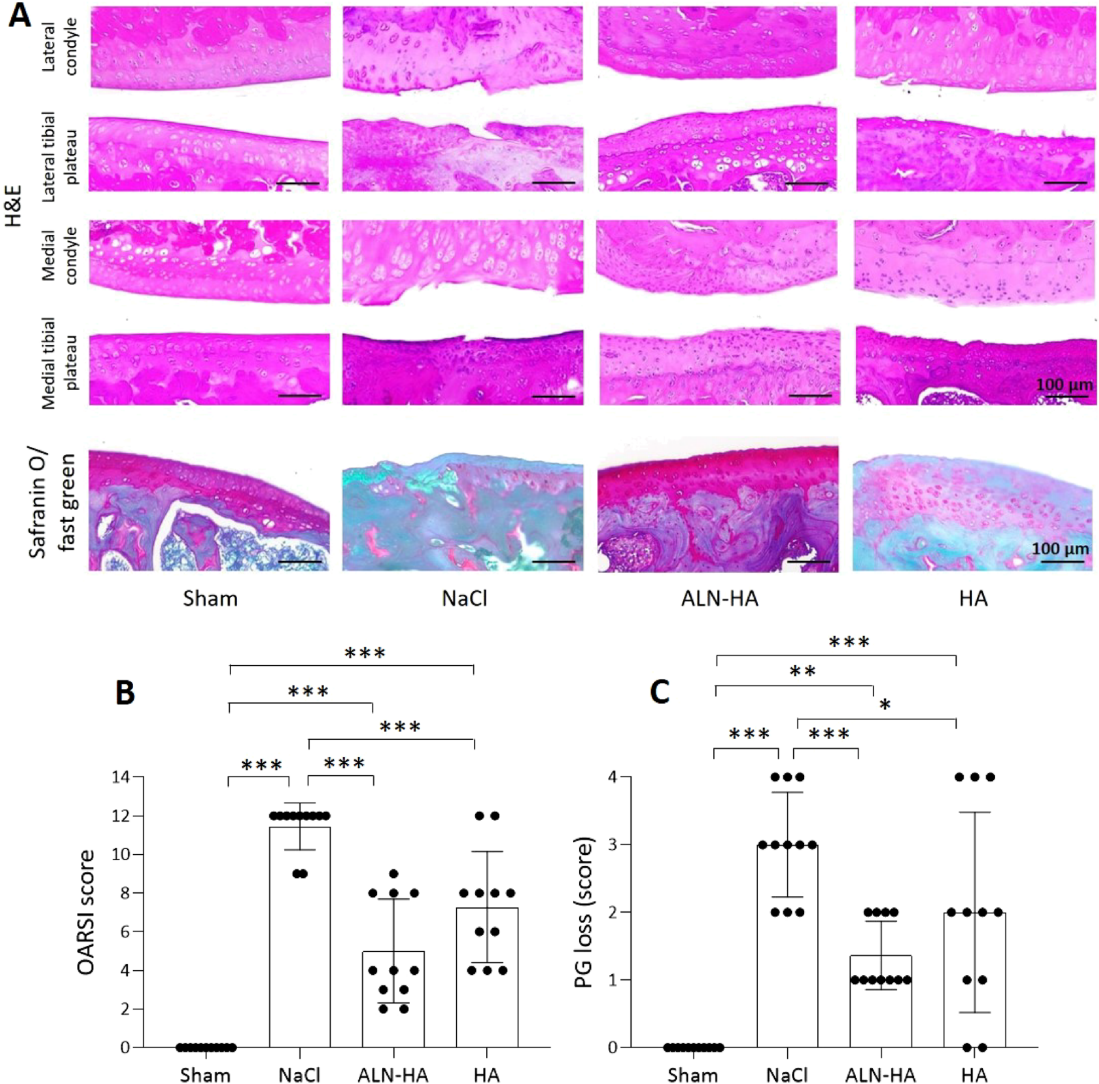
Histological evaluation of osteoarthritis. Histological analysis of the paw tissues was carried out at the end of the experiment. (**A**) Representative Hematoxylin and Eosin (H&E) and Safranin O/Fast Green-stained sections of the right knee joint of controls and NaCl, alendronate conjugate-hyaluronic acid (ALN-HA), and HA injected rats. Magnification 100×. (**B**) OARSI score and (**C**) proteoglycan score. Results are presented as mean ± SD of 11 rats per group. * < p0.05, **p < 0.01, ***p < 0.001.

Histological analysis showed the typical features of OA in NaCl-injected knees, including cartilage fibrillation and erosion with the presence of matrix cracks and fissures, loss of proteoglycan content, and decrease of the chondrocyte number. Knee joints treated with ALN-HA or HA highlighted a reduced erosion and fibrillation of the cartilage surface, and a greater chondrocyte number more orderly arranged in comparison to joint treated with NaCl. In addition, joints treated with ALN-HA were less rough without cracks, and showed an increase in cartilage staining compared to joints treated with HA (Fig. 3A). The presence of osteophytes was only slightly evident in the joints treated with NaCl. OARSI score highlighted that both ALN-HA and HA treated knee joints presented significantly lower values in comparison to NaCl (59.69% and 41.73%, respectively). In addition, OARSI score of ALN-HA group was 30.80% lower than that of HA (Fig. 3B). PG score showed significant decreased values in ALN-HA and HA injected knee joints compared to NaCl rats (54.54% and 33.33%, respectively). PG score of ALN-HA group was also 31.82% lower than that reported by the HA treated animals (Fig. 3C).

Synovial hypertrophy was observed in all samples from operated animals compared with the sham group. In rats treated with ALN-HA or HA the synovial membrane appeared more compact with a greater number of cells and vessels than in animals injected with NaCl, where instead it was highly disorganized.

### Effects of ALN-HA in cartilage biomarkers

**Figure 4:**
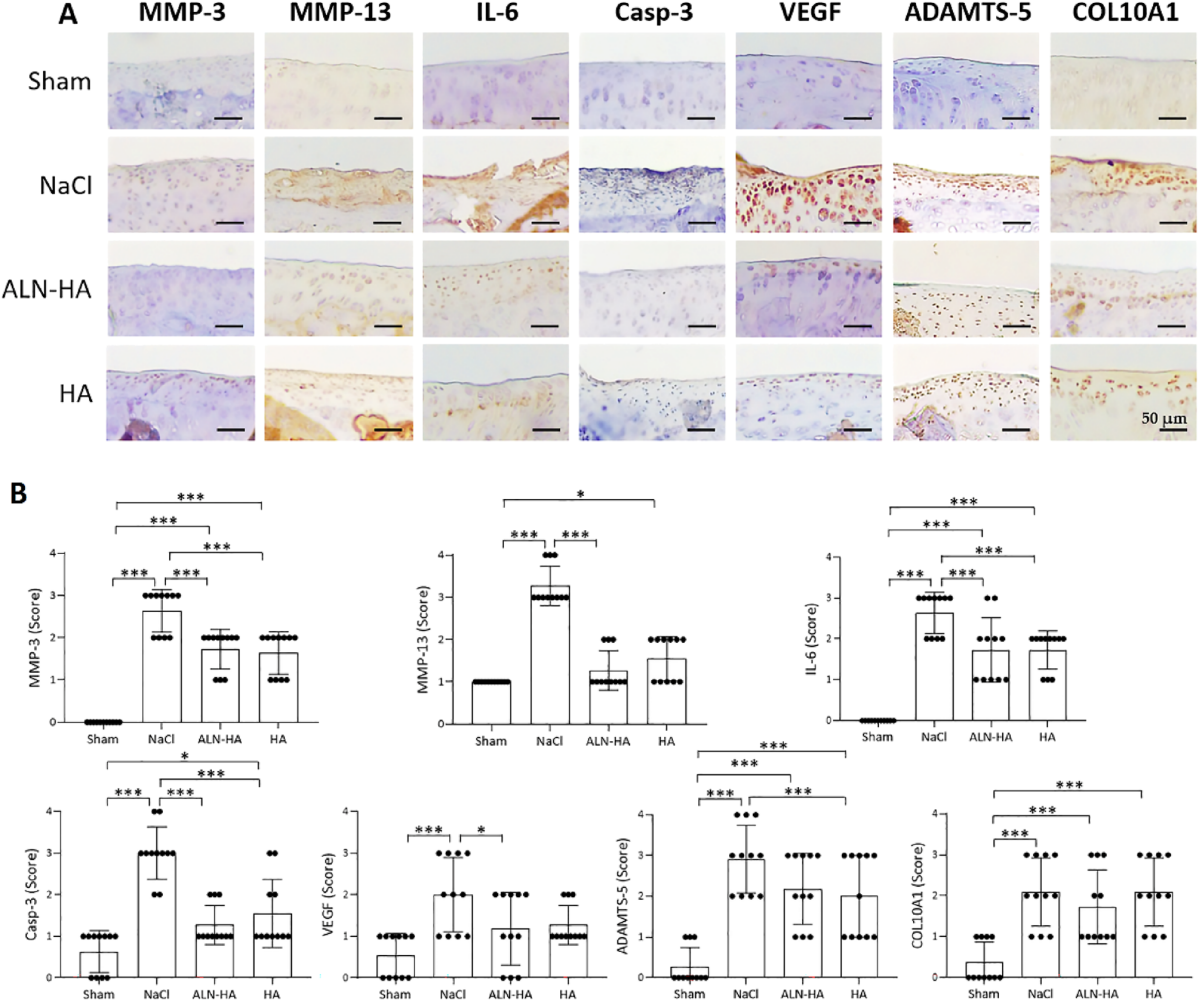
Immunohistochemistry analyses of articular cartilages. (**A**) Representative immunostaining of sections (magnification 100×) and (**B**) scoring of the right knee joint of controls and NaCl, alendronate conjugate-hyaluronic acid (ALN-HA), and HA injected rats. Results are presented as mean ± SD of 11 rats per group. * < p0.05, ***p < 0.001.

Representative pictures of immunohistochemical staining are reported in Fig. 4A. All considered markers were significantly more expressed in the cartilage of the rats subjected to DMM and treated with NaCl than in the sham group. MMP-13, MMP-3, IL-6 and Casp-3 scores were significantly decreased following both ALN-HA and HA alone treatment, compared with the NaCl group (Fig. 4B). VEGF score was significantly reduced only after ALN-HA treatment, while ADAMTS-5 score was significantly diminished after injection of HA alone. Of note, in the ALN-HA treated animals the levels of MMP-13, Casp-3 and VEGF were not significantly different than in the sham group. The COL10A1 score reported after ALN-HA or HA treatment were lower than those of the NaCl treated group, albeit not significantly. No significant changes were observed between ALN-HA and HA for any of the considered markers.

### Effects of ALN-HA in serum biomarkers

**Figure 5:**
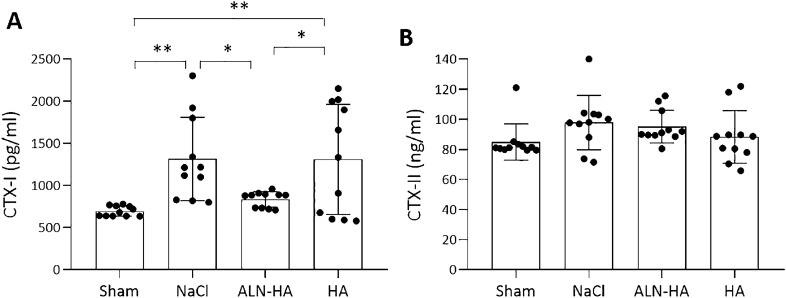
Serum concentration of Type (**A**) I and (**B**) II collagen cross-linked C-telopeptides (CTX-I and CTX-II) of controls and NaCl, alendronate conjugate-hyaluronic acid (ALN-HA), and HA injected rats. Results are presented as mean ± SD of 11 rats per group. * < p0.05, **p < 0.01.

NaCl group displayed the highest serum levels of CTX-I which were decresed after i.a. injection of ALN-HA, but not of HA alone, and brought back to non-pathological conditions (Fig. 5). No significant differences were found in CTX-II concentrations between groups.

## Discussion

This study demonstrated that a single i.a. injection of the conjugate ALN-HA attenuated OA development and progression in the rat DMM model, reducing pain, limiting cartilage damage and regulating matrix metabolism.

Pain is the primary symptom and a clinically relevant and important outcome measure of OA. Previous studies indicated that DMM is a standardized OA experimental model useful for pain research since it displays mechanical allodynia (a pain response induced by a stimulus that usually does not provoke pain)^39^. In this model, a decrease in withdrawal threshold determined by von Frey test is observed as early as few weeks after surgery, and is responsive to both paracetamol and morphine^40^. Our results show that, in rats subjected to DMM, allodynia occurred within the first weeks and persisted until the end of experiments in NaCl treated animals, as demonstrated by lower paw withdrawal threshold compared to sham group. Treatment with HA had no effect on pain, while a significative improvement was observed after ALN-HA injection. These results are in agreement with previous studies demonstrating that ALN displays pain modulation in OA. In particular, in a cross-sectional study involving elderly women with OA knee symptoms, ALN use was associated with significantly lower WOMAC pain scores compared with nonuse^41^. In addition, patients with OA knee taking bisphosphonate, predominantly ALN, reported a significant reduction in numerical rating scale (NRS) pain score during the first 3 years of observation when compared to bisphosphonate non-users^42^. It has been suggested that this analgesic benefit could be due to anti-inflammatory actions for BPs^43^. Indeed, a reduced expression of inflammatory cytokines, such as IL-6, IL-18, IL-1β, and TNF-α by inhibiting different signaling pathways has been observed after administration of ALN in several in vitro and in vivo models of arthritis and inflammation^43–46^, and a reduction of prostaglandin E2 levels in SF was correlated with the decrease of pain in OA patients after intra-articular repeated bisphosphonate administrations^47^.

Typical pathological features of OA include articular cartilage damage, subchondral bone changes, which can lead to osteophyte formation, and synovial hyperplasia and/or inflammation^48,49^. Currently, approved therapies for OA remain limited. Non-steroidal antiinflammatory drugs (NSAIDs), COX-2 inhibitors and steroids are the most commonly used drugs, but their overuse can induce serious side effects, and disease-modifying treatments are not available^7–9^. Intra-articular injection of HA remains the only recommended therapy after failure to respond to NSAIDs and in patients with contraindications to NSAIDs^50^, even though discrepancies exist among the different clinical practice guidelines^16^. Different preclinical and clinical data have proposed that ALN could act on subchondral bone turnover, synovitis and cartilage damage, although the results are mixed^19–21,51^, and recent clinical studies showed no benefit on pain or structure^52^. For instance, ALN subcutaneous injection reduced osteophyte formation and partially preserved articular cartilage in rat and mouse models of post-traumatic OA^19,24,29^. In contrast, Carbone et al. found no association of ALN use with changes in cartilage detected by MRI^41^. Our results showed that cartilage damage, determined by OARSI score, was significantly improved after administration of both HA and ALN-HA conjugate, thus confirming the chondroprotective effect of HA. Furthermore, the scores reported after ALN-HA treatment were lower than those of the HA treated group, thus suggesting that ALN may help in preserving articular cartilage and enhancing the action of HA. In our experiments, protection from cartilage degeneration was supported by immunohistochemical findings. Indeed, ALN-HA was found to decrease the expression of MMP-3, MMP-13 and ADAMTS5 which are key enzymes in degrading components of the extracellular matrix and play a critical role in OA progression^53–55^. This effect could be due to an anti-inflammatory action of the treatment mediated through a reduced production of pro-inflammatory cytokines. In line with this hypothesis, we observed in the same samples a lower expression of IL-6 and VEGF, which are potent inducers of MMPs and ADAMTS gene expression in articular chondrocytes^56,57^. Conversely, ALN-HA treatment did not show reduction of COL10A1 expression, a hypertrophic marker that increases during OA progression^58^.

Of note, only two previous research studies have explored the effect of i.a. injection of ALN in animal models of OA, showing reduction in cartilage tissue degeneration and loss of matrix proteins^59,60^. However, in both cases injection was performed in temporomandibular joint, whereas in our study ALN is administered for the first time into the knee. Therefore, we believe that i.a. injection could represent an effective option to ameliorate the major limitations of the use of BPs in the treatment of OA, thus allowing the administration of low doses of drugs and a direct action on the tissues involved in the disease; on the other hand, the lack of a control group treated with systemic ALN is a limitation of the study.

In our study, we found an improvement on the structure of the synovial membrane in ALN-HA and HA treated rats when compared to NaCl injected group, but we were unable to evaluate the effect of the formulations on the synovitis. This could be due to the fact that we performed histological analysis 12 weeks after disease induction in an experimental model of post-traumatic OA, where it was demonstrated that inflammation peaks in the early days after the injury^61,62^.

Interestingly, we also observed in cartilage of ALN-HA and HA treated animal a reduction of the expression of Casp-3, which is considered as a crucial mediator in inducing cell apoptosis^63^. These results are in agreement with previous studies demonstrating that both HA and ALN display anti-apoptotic properties in different in vitro and in vivo experimental models^64–67^.

Finally, we investigated the effects of i.a. ALN-HA or HA administration in systemic biomarkers of bone and cartilage turnover CTX-I and CTX-II. We observed a rise of CTX-I in injured rats that declines only after injection of the chemical conjugate containing ALN, thus confirming its ability to diminish increased bone resorption that occurs in OA. On the contrary, no significant differences were detected in CTX-II concentrations among all the experimental groups. This finding corroborates a previous study by Choi et al., who reported no associations between serum CTXII levels and histological assessment in DMM model in rats^68^.

## Conclusions

In conclusion, this study demonstrates that a single i.a. administration of the ALN-HA conjugate attenuates pain and progression of OA in rats and downregulates the expression of the main factors involved in the pathogenesis of the disease. The combined use of ALN and HA represents an interesting strategy for developing new potential interventions in the treatment of OA. Further studies should identify the exact mechanisms via which ALN alone or combined with HA exerts these analgesic and chondroprotective effects.

## Data Availability

The data used and analyzed during the current study are available from the corresponding author upon reasonable request.

## Abbreviations

ADAMTs: Anti-A Disintegrin And Metalloproteinase with Thrombospondin motifs
ALN: Alendronate
ALN-HA: Alendronate Hyaluronic Acid Conjugate
ALN-TBA: Alendronate Tetrabutylammonium salt
ANOVA: Analysis Of Variance
BMD: Bone Marrow Density
BP: Bisphosphonate
BV: Bone Volume
Casp: Caspase
COLX: Collagen-10
CTX-I: Type I Collagen cross-linked C-telopeptides
CTX-II: Type II Collagen cross-linked C-telopeptides
DAB: Diaminobenzidine
DMM: Destabilization of the Medial Meniscus
DMSO: Dimethylsulfoxide
ECM: Extra-Cellular Matrix
H&E: Hematoxylin and Eosin
HA: Hyaluronic Acid
HA-TBA: HA Tetrabutylammonium salt
i.a.: Intra-articular
IHC: Immunohistochemical
IL: Interleukin
MMP: Matrix Metalloproteinase
MMTL: Medial Meniscotibial Ligament
MRI: Magnetic Resonance Imaging
NRS: Numerical Rating Scale
NSAID: Non-Steroidal Anti-Inflammatory Drug
OA: Osteoarthritis
OARSI: Osteoarthritis Research Society International
PBS: Phosphate Buffered Saline
ROI: Region Of Interest
SF: Synovial Fluid
TGFβ: Transforming Growth Factor Beta
UPW: Ultra-Pure Water
VEGF: Vascular Endothelial Growth Factor
WOMAC: Western Ontario and McMaster Universities Osteoarthritis Index
